# Obinutuzumab and Ofatumumab are More Effective Than Rituximab in the Treatment of Membranous Nephropathy Patients With Anti-Rituximab Antibodies

**DOI:** 10.1016/j.ekir.2024.12.012

**Published:** 2024-12-17

**Authors:** Maxime Teisseyre, Marco Allinovi, Vincent Audard, Marion Cremoni, Giulia Belvederi, Alexandre Karamé, Matteo Accinno, Julien Duquesne, Vinod Sharma, Céline Fernandez, Kévin Zorzi, Mounir El Maï, Vesna Brglez, Sylvia Benzaken, Vincent L.M. Esnault, Alessandra Vultaggio, Harbir Singh Kohli, Raja Ramachandran, Calogero Lino Cirami, Barbara Seitz-Polski

**Affiliations:** 1 French Reference Center for Rare Diseases, Idiopathic Nephrotic Syndrome and Membranous Nephropathy, Nice University Hospital, Université Côte d’Azur, Nice, France; 2 Immunology Laboratory, Nice University Hospital, Université Côte d’Azur, Nice, France; 3 Department of Nephrology, Dialysis and Transplantation, Nice University Hospital, Université Côte d’Azur, Nice, France; 4 Unité de Recherche Clinique Côte d’Azur, Université Côte d’Azur, Nice, France; 5 Department of Nephrology, Dialysis and Transplantation, Careggi University Hospital, Florence, Italy; 6 French Reference Center for Rare Diseases, Idiopathic Nephrotic Syndrome and Membranous Nephropathy, Assistance Publique des Hôpitaux de Paris, Henri-Mondor University Hospital, Créteil, France; 7 Department of Nephrology, Dialysis and Transplantation, Assistance Publique des Hôpitaux de Paris, Henri-Mondor University Hospital, Créteil, France; 8 Paris Est Créteil University, Institut National de la Santé et de la Recherche Médicale U955, Institut Mondor de Recherche Biomédicale, Créteil, France; 9 Department of Nephrology and Dialysis, Néphropôle - Médipole Hôpital Privé, Lyon-Villeurbanne, Villeurbanne, France; 10 Department of Experimental and Clinical Medicine, University of Florence, Florence, Italy; 11 Department of Pharmacy, Nice University Hospital, Université Côte d’Azur, Nice, France; 12 Department of Nephrology, Postgraduate Institute of Medical Education and Research, Chandigarh, India; 13 Immunoallergology Unit, Careggi University Hospital, Florence, Italy

**Keywords:** antidrug antibodies, immunomonitoring, membranous nephropathy, obinutuzumab, ofatumumab, rituximab

## Abstract

**Introduction:**

Although rituximab has significantly improved outcomes for patients with membranous nephropathy, response to treatment is not universal and drug resistance can occur. One mechanism of resistance is the occurrence of antidrug antibodies. Obinutuzumab and ofatumumab are humanized and human monoclonal antibodies, respectively, that target B cells. These treatments have been shown to be effective in membranous nephropathy. However, obinutuzumab and ofatumumab have never been compared with rituximab in the treatment of patients with membranous nephropathy with anti-rituximab antibodies. We aimed to compare the efficacy and safety of obinutuzumab and ofatumumab with rituximab in patients with membranous nephropathy with anti-rituximab antibodies.

**Methods:**

This international retrospective multicenter study enrolled 34 patients with membranous nephropathy from 5 nephrology departments in France, India, and Italy. All the patients had previously developed anti-rituximab antibodies. Nineteen patients received rituximab, 12 received obinutuzumab, and 3 received ofatumumab.

**Results:**

Patients treated with obinutuzumab or ofatumumab were more likely to achieve clinical remission than those treated with rituximab at month 6 (87% vs. 37%, *P* = 0.005) and month 12 (87% vs. 42%, *P* = 0.01). Patients treated with obinutuzumab or ofatumumab were more likely to achieve immunological remission and B-cell depletion at month 6 than the patients treated with rituximab (92% vs. 56%, *P* = 0.04 and 93% vs. 35%, *P* = 0.002, respectively). No serious adverse events were reported in the obinutuzumab or ofatumumab group.

**Conclusion:**

Obinutuzumab and ofatumumab are more effective than rituximab in treating patients with membranous nephropathy with anti-rituximab antibodies. Anti-rituximab antibodies should be systematically monitored, to determine appropriate treatment.

## Graphical abstract

**Figure:**
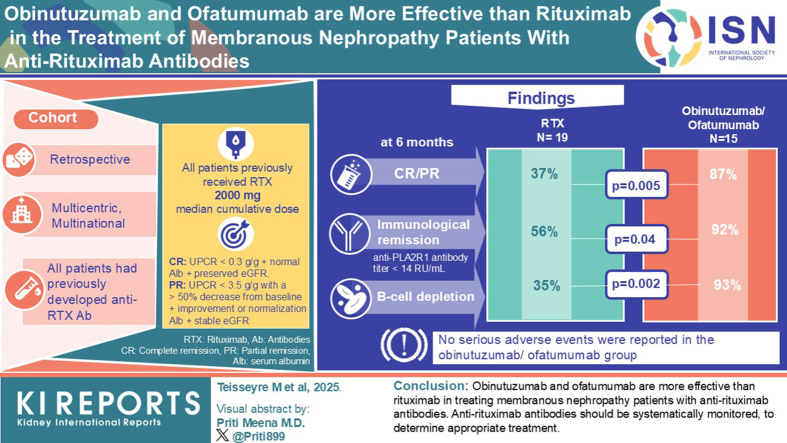

Primary membranous nephropathy is an autoimmune kidney disease resulting from autoantibodies. The main autoantibodies involved are directed against the phospholipase A2 receptor 1 (PLA2R1).^1^ The recognition of primary membranous nephropathy as an autoantibody-mediated disease has promoted the use of immunosuppressive drugs, such as rituximab. Rituximab is a chimeric IgG1 monoclonal antibody that targets CD20 on B cells, resulting in B-cell depletion and inhibition of autoantibody production. Rituximab is known to be a safe and effective treatment option for membranous nephropathy, achieving remission in 60%–80% of patients.^2^, ^3^, ^4^, ^5^, ^6^, ^7^ Therefore, rituximab is considered as one of the cornerstones of the therapeutic management of membranous nephropathy; however, the response to this treatment is not always satisfactory, and drug resistance may occur. The following 3 main factors may limit the efficacy of rituximab in membranous nephropathy: (i) chronic and irreversible glomerular damage, (ii) reduced bioavailability of rituximab, and (iii) the occurrence of anti-rituximab antibodies.^8^ The occurrence of antidrug antibodies is a well-known side effect after the use of monoclonal antibodies.^9^, ^10^, ^11^, ^12^, ^13^, ^14^, ^15^, ^16^, ^17^ Antidrug antibodies can alter the pharmacokinetic and pharmacodynamic properties of a treatment, thereby reducing its efficacy.^15^^,^^16^^,^^18^ In membranous nephropathy, rituximab immunization is a common concern, because 23% to 43% of patients treated with rituximab develop anti-rituximab antibodies during follow-up.^6^^,^^17^^,^^18^ Anti-rituximab antibodies can neutralize rituximab activity and are responsible for faster B-cell reconstitution, resulting in a higher relapse rate.^15^, ^16^, ^17^ This may also explain resistance to rituximab if present before treatment.^19^ We previously demonstrated that a high volume of distribution, a highly immunologically active disease (i.e., high anti-PLA2R1 antibody titer, PLA2R1 epitope spreading, and high interleukine-17A levels), and 25-hydroxyvitamin D deficiency are risk factors for the occurrence of anti-rituximab antibodies in patients with membranous nephropathy.^17^

Over time, a number of new anti-CD20 monoclonal antibodies, such as obinutuzumab and ofatumumab, have been developed. Obinutuzumab is a humanized and glycoengineered anti-CD20 IgG1 monoclonal antibody. Modification of the glycan tree structure in the Fc region results in increased affinity for FcγRIII, thereby enhancing antibody-dependent cellular cytotoxicity.^20^ Ofatumumab is a human anti-CD20 IgG1 monoclonal antibody. Ofatumumab activates complement-dependent cytotoxicity more effectively than rituximab.^21^ These humanized or human antibodies targeting CD20 have superior *in vitro* and *in vivo* cytotoxicity and a lower risk of immunogenicity than rituximab.^20^^,^^22^, ^23^, ^24^, ^25^, ^26^ Obinutuzumab and ofatumumab have been shown to be effective in patients with membranous nephropathy.^19^^,^^27^, ^28^, ^29^, ^30^, ^31^, ^32^, ^33^ However, obinutuzumab and ofatumumab have never been compared with rituximab in the treatment of patients with membranous nephropathy who have developed anti-rituximab antibodies after previous exposure to rituximab. Here, we aimed to compare rituximab with obinutuzumab and ofatumumab in the treatment of patients with primary membranous nephropathy with anti-rituximab antibodies.

## Methods

### Study Participants

This is an international multicenter, retrospective, observational study conducted in the following 5 nephrology centers: Chandigarh, India; Créteil, France; Florence, Italy; Nice, France; and Villeurbanne, France. Inclusion criteria were as follows: (i) membranous nephropathy diagnosed by using renal biopsy or serological assay; (ii) primary membranous nephropathy defined by the absence of concomitant autoimmune disease, negative human immunodeficiency virus, hepatitis B and C serologies, and negative cancer workup; and (iii) pretreatment detection of anti-rituximab antibodies.

Patients were assigned to a specific treatment (rituximab, ofatumumab, or obinutuzumab) based on the center's standard practice and local treatment availability. The study protocol conformed to the ethical guidelines of the 1975 Declaration of Helsinki. The local ethics committees approved the study. Patients provided informed consent for treatment with anti-CD20 monoclonal antibodies.

### Outcome

Clinical remission was assessed at month 6 and month 12. Clinical remission was defined according to the Kidney Disease Improving Global Outcomes guidelines.^34^ Complete remission was defined as a urine protein-to-creatinine ratio < 0.3 g/g, accompanied by a normal serum albumin level and a preserved renal function. Partial remission was defined as urine protein-to-creatinine ratio < 3.5 g/g with a > 50% decrease from baseline, accompanied by an improvement or normalization of the serum albumin level and stable renal function. Patients who did not meet remission criteria or who received new treatment were considered as treatment failure.

Relapse was defined as a recurrence of nephrotic syndrome after a clinical remission phase.

Immunological remission was defined as an anti-PLA2R1 antibody titer < 14 RU/ml by using enzyme-linked immunosorbent assay.

Rituximab resistance was defined as the absence of clinical and immunological remission at month 6 after rituximab administration or absence of clinical remission at month 9 after rituximab administration.

### B-Cell Count

B-cell depletion was defined as a CD19^+^ cell count < 5/mm^3^

### Anti-PLA2R1 Antibodies

Anti-PLA2R1 antibody titers were measured by using the EUROIMMUN enzyme-linked immunosorbent assay kit (Medizinische Labordiagnostika AG, Lübeck, Germany). The limit of detection was 14 RU/ml.

### Anti-Rituximab Antibodies

Anti-rituximab antibodies were detected by LISA-TRACKER enzyme-linked immunosorbent assay (Theradiag, Croissy Beaubourg, France). The limit of detection defined by the manufacturer was 5 ng/ml.

### Adverse Events

Safety parameters included all serious and nonserious adverse events and unexpected changes in clinical or laboratory parameters, observed throughout the follow-up period.

### B-Cell Cytotoxicity Assay

After a Ficoll separation, 1.8 × 10^6^ peripheral blood mononuclear cells from healthy donors were preincubated with 20 μl of serum from 2 patients, with anti-rituximab antibodies diluted 1:2 or phosphate-buffered saline as control, then incubated overnight at 37 °C with rituximab at 50 ng/ml, obinutuzumab at 50 ng/ml, or phosphate-buffered saline as control. The cells were washed 3 times and incubated 30 minutes in darkness with anti-CD3, anti-CD45, and anti-CD19 antibodies (BD Biosciences, Franklin Lakes, NJ), and then fixed. The percentages of B cells (CD19^+^) were determined using BD FACSLyric flow cytometry system (BD Biosciences, Franklin Lakes, NJ). The different conditions were expressed as a percentage of B cells compared with the standard condition (without patient serum or anti-CD20 antibodies).

### Statistical Analyses

Categorical data were described as frequencies and percentages and were compared using Fisher exact test or chi-square test. Quantitative data were described as median and interquartile range and were compared using the Wilcoxon–Mann–Whitney test. Survival curves were assessed by the Kaplan–Meier method and compared with the log-rank test. All tests were 2-tailed and a *P*-value < 0.05 was considered statistically significant. Statistical analyses were performed with GraphPad Prism 8.4.3 (GraphPad Software Inc., San Diego, CA).

## Results

Thirty-four patients with both primary membranous nephropathy and anti-rituximab antibodies were enrolled. In Table 1, we describe the baseline characteristics of the patients. Nineteen patients were treated with rituximab and received 1000 mg on day 1 and day 15 (except for 1 patient who received a 1st dose of 1000 mg but did not attend his 2nd injection on day 15, and it was decided to restart the protocol for him with 2 new injections of 1000 mg, 2 weeks apart on days 30 and 45). Twelve patients were treated with obinutuzumab and received 100 mg on day 1, 900 mg on day 2, and 1000 mg on days 8 and 15 (i.e., a cumulative dose of 3000 mg). Three patients were treated with ofatumumab and received 300 mg on day 1 and 1000 mg on day 8 (except for 1 patient who received 300 mg on day 1, and 1000 mg on days 8 and 21). All patients were previously treated with rituximab with a median cumulative dose of 2000 [2000–4000] mg. The median time from last treatment to current treatment was 12 [8–27] months. At the time of enrollment, 20 patients (10 in each group) were considered resistant to rituximab, and the remaining 14 were treated for clinical relapse. Median follow-up was 96 [52–124] weeks in the obinutuzumab or ofatumumab group and 72 [48–98] weeks in the rituximab group (*P* = 0.2).

**Table 1:** Patients’ characteristics

Characteristics at baseline	All patients (*n* = 34)	Rituximab (*n* = 19)	Obinutuzumab or ofatumumab (*n* = 15)	*P*–value
Gender (male/female)	16/18	9/10	7/8	1
Age (yrs), median [Q1–Q3]	62 [49–72]	67 [50–74]	57 [46–69]	0.7
Weight (kg), median [Q1–Q3]	75.0 [65.9–93.5]	71.0 [61.1–93.3]	78.0 [70.0–103.0]	0.3
BMI (kg/m^2^), median [Q1–Q3]	27.6 [24.4–31.1]	27.5 [23.9–31.2]	28.9 [24.9–30.9]	0.8
Serum albumin (g/l), median [Q1–Q3]	26.0 [22.3–31.1]	30.0 [23.2–32.0]	25.2 [22.4–30.0]	0.3
UPCR (g/g), median [Q1–Q3]	5.8 [4.2–7.5]	5.7 [4.1–7.6]	5.9 [4.7–7.5]	0.9
eGFR (ml/min per 1.73 m^2^), median [Q1–Q3]	60 [27–78]	60 [18–74]	59 [29–83]	0.8
Anti-PLA2R1–associated membranous nephropathy, *n* (%)	30 (88%)	17 (89%)	13 (87%)	1
Anti-PLA2R1 Ab titer (RU/ml), median [Q1–Q3]	60 [25–235]	104 [25–296]	50 [27–217]	0.7
Anti-rituximab Ab titer (ng/ml), median [Q1–Q3]	20 [10–49]	14 [9–47]	28 [12–55]	0.4
Previous rituximab treatment (cumulative dose in g), median [Q1–Q3]	2 [2–4]	2 [2–3]	3 [2–4]	0.08
Previous rituximab treatment (months since last cure), median [Q1–Q3]	12 [8–27]	13 [7–28]	12 [9–26]	0.8
Rituximab resistance, *n* (%)	20 (59%)	10 (53%)	10 (67%)	0.5

Ab, antibody; BMI, body mass index; CKD-EPI, Chronic Kidney Disease-Epidemiology Collaboration formula; eGFR, estimated glomerular filtration rate (CKD-EPI); PLA2R1, phospholipase A2 receptor 1; Q1, first quartile 1; Q3, third quartile; UPCR, urine protein-to-creatinine ratio.

**Figure 1:**
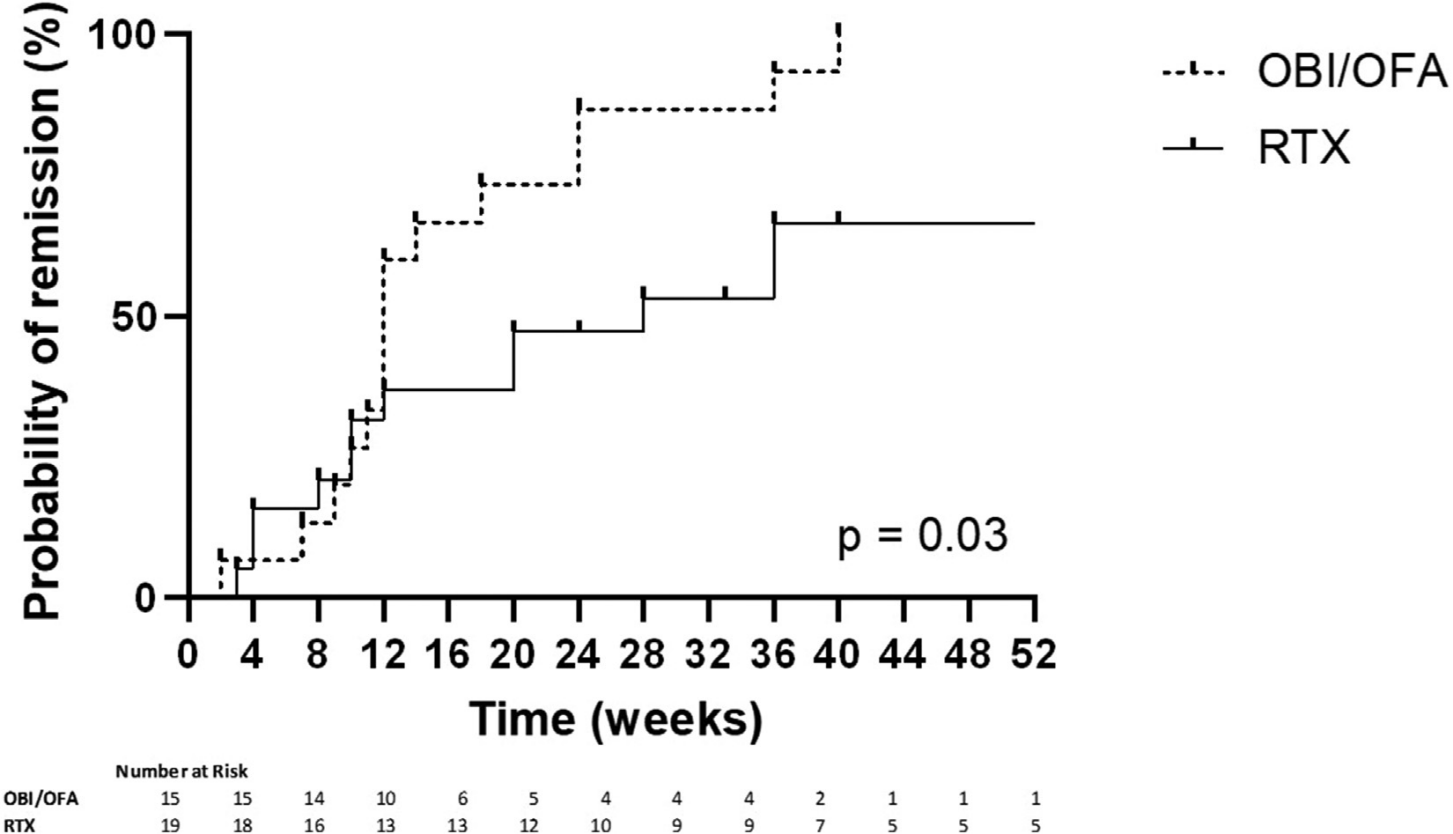
Time to clinical remission. Kaplan–Meier estimate of clinical remission (complete or partial) in the obinutuzumab or ofatumumab group versus rituximab group. OBI/OFA, obinutuzumab or ofatumumab group; RTX, rituximab group.

Patient outcomes are summarized in Table 2. We found that patients in the obinutuzumab or ofatumumab group were more likely to achieve clinical remission over time (*P* = 0.03) (Figure 1).

**Table 2:** Patient Outcome

Outcome	Rituximab	Obinutuzumab or ofatumumab	*P*-value
	Number of patients/total number (%)		
Month 6			
Clinical remission^a^	7/19 (37%)	13/15 (87%)	0.005
Immunological remission^b^^,^^c^	9/16 (56%)	12/13 (92%)	0.04
B-cell depletion^d^	6/17 (35%)	13/14 (93%)	0.002
Anti-rituximab antibody positivity^e^	4/13 (31%)	5/10 (50%)	0.4
Month 12			
Clinical remission^a^	8/19 (42%)	13/15 (87%)	0.01
Immunological remission^b^^,^^d^	7/15 (47%)	10/12 (83%)	0.1
B cell depletion^f^	0/2 (0%)	4/12 (33%)	1
Anti-rituximab antibody positivity^g^	6/12 (50%)	4/9 (44%)	1

a Partial clinical remission was achieved at months 6 and 12 in the 3 patients treated with ofatumumab.

b Among patients with PLA2R1-associated membranous nephropathy.

c One missing value for the rituximab group.

d Two missing values for the rituximab group, and 1 missing value for the obinutuzumab or ofatumumab group.

e Six missing values for the rituximab group, and 5 missing values for the obinutuzumab or ofatumumab group.

f Seventeen missing values for the rituximab group, and 3 missing values for the obinutuzumab or ofatumumab group.

g Seven missing values for the rituximab group, and 6 missing values for the obinutuzumab or ofatumumab group.

At month 6, 13 patients (87%) in the obinutuzumab or ofatumumab group achieved clinical remission (partial or complete) versus 7 patients (37%) in the rituximab group (*P* = 0.005). One patient in the obinutuzumab or ofatumumab group achieved a complete clinical remission versus none in the rituximab group. Patients treated with obinutuzumab or ofatumumab were more likely to achieve B-cell depletion at month 6 (35% in the rituximab group vs. 93% in the obinutuzumab or ofatumumab group, *P* = 0.002). Among the patients with PLA2R1-associated membranous nephropathy, 12 patients (92%) in the obinutuzumab or ofatumumab group and 9 patients (56%) in the rituximab group achieved immunological remission at month 6 (*P* = 0.04) (Table 2). At month 12, 13 patients (87%) in the obinutuzumab or ofatumumab group achieved clinical remission (partial or complete) versus 8 patients (42%) in the rituximab group (*P* = 0.01). One patient in the obinutuzumab or ofatumumab group achieved complete clinical remission versus 2 patients in the rituximab group. Among patients in the rituximab group, those who achieved clinical remission at month 12 had significantly higher baseline proteinuria than those who did not achieve clinical remission (Supplementary Table S2). Among patients with PLA2R1-associated membranous nephropathy, 10 patients (83%) in the obinutuzumab or ofatumumab group and 7 patients (47%) in the rituximab group achieved immunological remission at month 12 (*P* = 0.1) (Table 2). The median time to remission was similar between the 2 groups (12 months in the obinutuzumab or ofatumumab group versus 11 months in the rituximab group, *P* = 0.7). Three patients in the rituximab group were retreated before month 12, whereas none in the obinutuzumab or ofatumumab group were retreated before the same period.

At the time of enrollment, 10 patients in both groups were considered resistant to previous rituximab treatment. After treatment, 8 patients (80%) in the obinutuzumab or ofatumumab group versus 3 (30%) in the rituximab group achieved clinical remission at month 6 (*P* = 0.07); and 9 patients (90%) in the obinutuzumab or ofatumumab group versus 3 (30%) in the rituximab group achieved clinical remission at month 12 (*P* = 0.02). The rituximab-resistant patient who did not respond to obinutuzumab finally achieved clinical remission after a new course of obinutuzumab.

Among patients who achieved clinical remission, the relapse rate at the last follow-up was not statistically different between groups (33% in the obinutuzumab or ofatumumab group vs. 42% in the rituximab group, *P* = 0.7). Relapse occurred at a median of 124 [37–214] weeks in the obinutuzumab or ofatumumab group versus 52 [46–66] weeks in the rituximab group (*P* = 0.7).

The prevalence of anti-rituximab antibodies at months 6 and 12 was similar between the 2 groups (Table 2).

One patient treated with ofatumumab and 2 patients treated with rituximab experienced infusion-related reactions, which were resolved by reducing the treatment flow rate. Two patients treated with rituximab developed upper respiratory tract infections, which were treated successfully with favorable outcomes. One patient developed a urinary tract infection 9 months after treatment with ofatumumab, which was cured after antibiotic treatment and a 4-day hospital stay. One patient in each group reported digestive disorders such as nausea and diarrhea after treatment. One patient in the rituximab group died at month 6 from an unrelated cause. No serious adverse events were reported in the obinutuzumab or ofatumumab group. The patient who received the highest cumulative dose of rituximab developed a severe aspergillosis infection requiring hospitalization in an intensive care unit. Adverse events are summarized in Supplementary Table S1.

**Figure 2:**
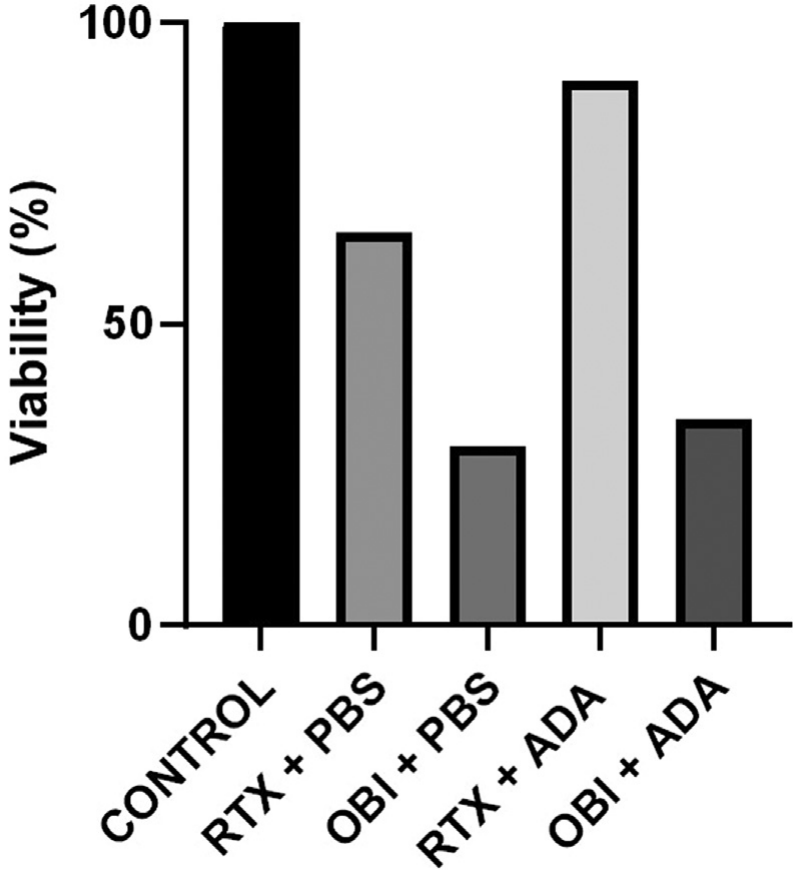
B-cell cytotoxicity assay. Peripheral blood mononuclear cells (1.8 × 10^6^) from healthy donors were preincubated with 20 μl of serum from 2 patients with anti-rituximab antibodies diluted 1:2 (ADA) or PBS as a control, then incubated overnight at 37 °C with rituximab 50 ng/ml, obinutuzumab 50 ng/ml, or PBS as a negative control. The percentage of B cells (CD19^+^) was determined by flow cytometry. The different conditions were expressed as percentage of B cells (i.e., viability) compared with the control condition (without patient serum or anti-CD20 antibody). ADA, serum with antidrug antibodies; OBI, obinutuzumab; PBS, phosphate-buffered saline; RTX, rituximab.

*In vitro,* obinutuzumab was more effective than rituximab at the same dose in inducing B-cell depletion. The efficacy of obinutuzumab was maintained in the presence of anti-rituximab antibodies, unlike rituximab (Figure 2), which is consistent with other studies showing that anti-rituximab antibodies rarely, if ever, cross-reacted with obinutuzumab or ofatumumab.^15^^,^^18^

## Discussion

In this study, we retrospectively compared treatment with obinutuzumab or ofatumumab versus rituximab in patients with membranous nephropathy who previously developed anti-rituximab antibodies. According to the 2021 Kidney Disease Improving Global Outcomes Guidelines, evaluating the clinical impact of anti-rituximab antibodies, and investigating the efficacy of new treatments targeting B cells in patients with membranous nephropathy refractory to standard therapy are research priorities.^34^

Understanding the mechanisms of resistance to rituximab is an important issue in the management of membranous nephropathy to personalize treatment.^8^ Some patients with membranous nephropathy and severe nephrotic syndrome are undertreated because of the loss of the drug in the urine.^35^^,^^36^ In these patients, additional doses of rituximab could increase the likelihood of remission.^37^, ^38^, ^39^ Therefore, individualization of the rituximab dose is critical. To this end, we have recently developed an algorithm to estimate the risk of rituximab underdosing based on baseline clinical and biological parameters.^38^ A study is underway to evaluate the initial adjustment of rituximab doses (from 2000 mg in 2 infusions to 4000 mg in 4 infusions) as per the risk of underdosing estimated by the algorithm compared with the conventional regimen (iRITUX trial, NCT06341205). Another common mechanism of resistance is the development of anti-rituximab antibodies. The development of anti-rituximab antibodies has been described in several autoimmune diseases following rituximab treatment, with variable outcomes.^16^^,^^40^, ^41^, ^42^, ^43^, ^44^, ^45^, ^46^ In childhood nephrotic syndrome, anti-rituximab antibodies are detected in 29% to 38% of patients and are associated with a decreased B-cell depletion and a higher risk of relapse.^42^^,^^46^ Anti-rituximab antibodies are also frequently detected in membranous nephropathy and are associated with a poorer outcome.^15^^,^^17^ In such cases, humanized or human monoclonal antibodies offer a viable alternative. Unlike rituximab, human or humanized monoclonal antibodies appear to be less likely to induce drug immunization.^26^^,^^47^^,^^48^ In autoimmune or inflammatory diseases, humanized or human monoclonal antibodies have proven effective after the emergence of resistance to chimeric monoclonal antibodies because of the appearance of antidrug antibodies.^49^, ^50^, ^51^ In frequently relapsing or steroid-dependent nephrotic syndrome in children, obinutuzumab has been shown to be effective in cases of resistance or relapse after rituximab treatment.^52^ Several case series have shown that obinutuzumab or ofatumumab are effective in inducing clinical remission in rituximab-resistant membranous nephropathy.^19^^,^^27^, ^28^, ^29^, ^30^, ^31^, ^32^ All but one of these series did not monitor anti-rituximab antibodies.^19^ In a recent study, obinutuzumab was superior to rituximab in inducing clinical remission in patients with membranous nephropathy.^33^ Several other prospective studies are underway evaluating obinutuzumab in membranous nephropathy (NCT04629248 and NCT06120673), particularly in cases resistant to rituximab (NCT05050214 and NCT05845762). However, anti-rituximab antibody monitoring has not been used to personalize treatment in any of these studies. Personalizing treatment according to the mechanism of resistance would limit costs while improving efficacy.

Here, we have shown that obinutuzumab and ofatumumab are significantly more effective than rituximab in inducing clinical and immunological remission in adult patients with membranous nephropathy who have developed anti-rituximab antibodies, while remaining safe. The superiority of obinutuzumab and ofatumumab may be explained by the fact that anti-rituximab antibodies do not cross-react with these drugs, thus improving B-cell depletion.^15^^,^^18^ Our study supports this hypothesis because we have shown that patients treated with obinutuzumab or ofatumumab were more likely to achieve B-cell depletion and immunological remission at month 6. The relapse rate was similar between groups, but the median follow-up time was shorter in the rituximab group and remission was less frequent in the rituximab group, which may underestimate the relapse rate in this group. The time to relapse was more than twice as long in the obinutuzumab or ofatumumab group, although this was not significant, probably because of lack of power.

Notwithstanding the presence of anti-rituximab antibodies, a subset of patients in the rituximab group achieved clinical remission. We propose 2 hypotheses. First, anti-rituximab antibodies may in some cases not completely neutralize the effect of rituximab.^15^ Second, in our cohort, among patients in the rituximab group, those who achieved clinical remission at month 12 had significantly higher baseline proteinuria than those who did not achieve clinical remission. Proteinuria may promote urinary drug loss and, together with the neutralizing effect of anti-rituximab antibodies, further limit rituximab exposure. This may suggest that a higher cumulative dose of rituximab, particularly in patients with high proteinuria, may improve response to treatment, even in the presence of anti-rituximab antibodies. However, in patients immunized against rituximab, reexposure to the drug could enhance immunization against the treatment and thus alter the long-term prognosis of the patient.

These results indicate that monitoring for anti-rituximab antibodies in membranous nephropathy following rituximab treatment is of particular concern and should be performed systematically, especially in cases of relapse or rituximab resistance, to determine more appropriate treatment.

This study has several limitations. First, it is a retrospective study; however, it is the first international multicenter study to compare rituximab with human or humanized anti-CD20 therapies in patients with membranous nephropathy and anti-rituximab antibodies. Second, the number of participants is relatively small; however, this is a rare disease and monitoring for anti-rituximab antibodies is a recent technique that is not yet routinely performed in all centers. Third, the anti-PLA2R1 antibody titer was higher in the rituximab group than in the obinutuzumab or ofatumumab group, although this was not statistically significant. In addition, the severity of nephrotic syndrome was greater in the obinutuzumab or ofatumumab group than in the rituximab group (serum albumin 25.2 vs. 30.0 g/l and urine protein-to-creatinine ratio 5.9 vs. 5.7 g/g), although this was not statistically significant. Finally, the dose of obinutuzumab used was higher than that of rituximab, although this does not fully explain the higher efficacy, as we have shown that obinutuzumab was more effective *in vitro* than rituximab at the same dose in inducing B-cell depletion in the presence of anti-rituximab antibodies.^15^ In addition, 2 patients were treated with a lower dose of ofatumumab than rituximab with remissions at 6 and 12 months, and 1 patient was treated with 3000 mg of rituximab without efficacy. It is important to note that these treatments are different drugs for which there is currently no harmonized treatment protocol. Dosages of ofatumumab and obinutuzumab were chosen based on those used in hematological malignancies.^53^, ^54^, ^55^, ^56^ The first injection was given at a lower dose in order to reduce infusion reactions.

In conclusion, obinutuzumab and ofatumumab are more effective than rituximab in the treatment of patients with membranous nephropathy and anti-rituximab antibodies. Monitoring of anti-rituximab antibodies should be performed routinely in patients with membranous nephropathy after treatment with rituximab, especially in the event of relapse or rituximab resistance, to personalize treatment. These results should be confirmed in a prospective multicenter study with a larger number of patients. Further studies are needed to propose a harmonized regimen for obinutuzumab and ofatumumab in membranous nephropathy.

## Disclosure

All the authors declared no competing interests.

## Data Availability Statement

The raw data supporting the conclusions of this article are available from the authors upon request.

## Author Contributions

MT, MA, CLC, and BS-P contributed to the conception and design of the study. MT and MA contributed to the analysis and interpretation of data. All the authors participated in drafting of the article and approved the final version submitted.

## Supplementary Material

Supplementary File (PDF) **Table S1**. Adverse events according to treatment group. **Table S2**. Characteristics of patients in the rituximab group according to treatment response at month 12.

## Footnotes

Supplementary File (PDF)
**Table S1**. Adverse events according to treatment group. **Table S2.** Characteristics of patients in the rituximab group according to treatment response at month 12.
