# Supplementary material for: Obinutuzumab and Ofatumumab are More Effective Than Rituximab in the Treatment of Membranous Nephropathy Patients With Anti-Rituximab Antibodies
Source: Kidney Int Rep. 2024 Dec 17;10(3):753–61. doi: 10.1016/j.ekir.2024.12.012 (PMC11993203; doi:10.1016/j.ekir.2024.12.012)
Supplement: Supplementary File (PDF) — Table S1. Adverse events according to treatment group. Table S2. Characteristics of patients in the rituximab group according to treatment response at month 12. [file mmc1.pdf]

Table S1. Adverse events according to treatment group

Adverse events	Rituximab	Obinutuzumab/ofatumumab	*p-value*
	number of patients/total number (%)		
Infusion-related reaction	2/19 (11%)	2/15 (13%)	1
Infection	3/19 (16%)	1/15 (7%)	0.6
Urinary tract infection	0/19 (0%)	1/15 (7%)	
Upper respiratory infection	2/19 (10%)	0/15 (0%)	
Severe infection	1/19 (5%)	0/15 (0%)	
Others			
Digestive disorders	1/19 (5%)	1/15 (7%)	1
Pain	0/19 (0%)	1/15 (7%)	0.4

Table S2. Characteristics of patients in the rituximab group according to treatment response at month-12

Characteristics at baseline	Clinical remission (n=8)	No clinical remission (n=11)	*p*-value
Sex (male/female)	2/6	7/4	0.2
Age (years)	62 [44-72]	67 [51-76]	0.6
Weight (kg)	71.8 [63.0-85.0]	70.0 [60.0-94.0]	0.9
BMI (kg/m^2)	27.1 [24.2-31.2]	27.5 [23.9-32.7]	0.9
Serum albumin (g/L)	30.3 [25.0-33.9]	26.0 [22.0-31.5]	0.3
UPCR (g/g)	5.0 [4.0-5.7]	7.0 [4.2-11.1]	0.048
eGFR (mL/min/1.73m^2)	70 [40-91]	44 [16-73]	0.3
Anti-PLA2R1 titer (RU/mL)	207 [19-476]	104 [25-210]	0.5
Anti-rituximab Ab titer (ng/mL)	14.6 [9.5-76.3]	14.0 [6.0-40.0]	0.5

Abbreviations: Ab, antibody; BMI, body mass index; eGFR, estimated glomerular filtration rate (CKD-EPI); PLA2R1, phospholipase A2 receptor 1; Q1, first quartile 1; Q3, third quartile; UPCR, urine protein-creatinine ratio
